# Preoperative management of antiplatelet drugs for a coronary artery stent: how can we hit a moving target?

**DOI:** 10.1186/1471-2253-14-73

**Published:** 2014-08-23

**Authors:** Thomas R Vetter, James M Hunter, Arthur M Boudreaux

**Affiliations:** 1 Department of Anesthesiology, School of Medicine, University of Alabama at Birmingham, 619 19th Street South, JT804, Birmingham, AL 35249-6810, USA

**Keywords:** Coronary artery stent, Antiplatelet therapy, Preoperative, Major adverse cardiac event, Standardized clinical assessment and management plan, Consensus-oriented decision-making model

## Abstract

**Background:**

With the advent of percutaneous coronary intervention, specifically the bare metal stent and subsequently, the drug-eluting stent, the scope of interventional cardiology has greatly increased. Aspirin, in combination with a thienopyridine is the present-day cornerstone of oral antiplatelet therapy after coronary artery stent placement. Continuing this chronic antiplatelet therapy, to mitigate a perioperative major adverse cardiac event, can be challenging and remains controversial in patients with a coronary artery stent undergoing non-cardiac surgery. We describe here the rationale for and successful use of an alternate approach to formulating local institutional management protocols for patients with a coronary artery stent, undergoing an elective surgical procedure.

**Discussion:**

A recent systematic review identified 11 clinical practice guidelines for the perioperative management of antiplatelet therapy in patients with a coronary stent who need non-cardiac surgery. However, there is significant variance and inadequacy with these current applicable professional society guidelines. Moreover, persistently variable success has been experienced in translating even well-grounded national clinical guidelines into local practice, including in the perioperative setting. Under the auspices of a broadly multidisciplinary institutional task force and applying the Consensus-Oriented Decision-Making model, we created two evidence-informed and local expert opinion-supported standardized clinical assessment and management plans for the preoperative management of antiplatelet therapy in patients with a coronary artery stent.

**Summary:**

Patient care can be optimized via evidence-based, yet locally developed and reiterative standardized clinical assessment and management plans for patients with coronary artery stents undergoing surgical procedures. Such standardized clinical assessment and management plans can result in greater consistency in care, providing a positive feedback loop in which the care plan itself can be continuously reevaluated, improved, and brought up to date with the most recent available data and knowledge.

## 

“Success is simple. Do what’s right, the right way, at the right time.”

Arnold H. Glasgow (1905-1998), American Businessman and Humorist

## Background

With the advent of percutaneous coronary intervention (PCI), specifically the bare metal stent (BMS) and subsequently, the drug-eluting stent (DES), the scope of interventional cardiology has greatly increased [1-3]. An estimated 600,000 coronary artery stents are placed annually in the United States (US) for the management of acute and chronic coronary artery disease [4]. Given the aging US population and its increasing prevalence of coronary artery disease, the use of coronary artery stents will likely continue to grow. These biomedical devices appear to have reduced the number of more invasive coronary artery bypass surgeries [1,4]. However, US Medicare expenditures for drug-eluting stents alone (not including the cost of chronic antiplatelet drugs) are currently estimated to be $1.57 billion per year and are expected to increase [5].

Aspirin, in combination with a thienopyridine (e.g., clopidogrel), is the present-day cornerstone of oral antiplatelet therapy for the prevention of acute stent thrombosis, after placement of a BMS or a DES [6,7]. The cumulative incidence of non-cardiac surgery following coronary artery stenting is more than 10% at one year and over 20% at two years [8]. Continuing this chronic antiplatelet therapy, to mitigate a perioperative major adverse cardiac event (MACE), can be challenging and remains controversial in patients with a coronary artery stent undergoing non-cardiac surgery [9-11]. While applicable evidence-based guidelines have been promulgated, we observed that they can lack timeliness, clarity, and applicability—resulting in variation in actual use. We thus describe here the rationale for and successful use of an alternate approach to formulating local institutional management protocols for patients with a coronary artery stent undergoing an elective surgical procedure.

### Existing Guidelines for the Perioperative Management of Antiplatelet Therapy in Patients with a Coronary Artery Stent

A recent systematic review identified 11 clinical practice guidelines for the perioperative management of antiplatelet therapy in patients with a coronary stent who need non-cardiac surgery [12]. These authors applied the Appraisal of Guidelines Research and Evaluation II (AGREE II) instrument (with a maximum score of 161) to assess the quality of the identified guidelines [12], Five of the 11 practice guidelines had an AGREE II score of > 100: American College of Chest Physicians [13], American College of Cardiology/ American Heart Association (ACC/AHA) [14], Canadian Cardiovascular Society [15], European Society for Cardiology [16], and Institute for Clinical Systems Improvement [17]. The Society of Thoracic Surgeons has also published updated guidelines on the management of antiplatelet therapy in cardiac and non-cardiac surgical patients [18].

However, despite some having AGREE II scores of greater than 100, a paucity of available definitive evidence has resulted in perioperative coronary artery stent guidelines that are divergent and vague on key issues. Recommendations vary regarding the amount of time which must elapse between stent placement and elective surgery in order to have an acceptable risk of acute stent thrombosis when dual antiplatelet therapy (DAPT) is discontinued. If DAPT must be interrupted within this critical period due to the risk of bleeding, the recommended timing of aspirin and thienopyridine discontinuation also varies.

These existing guidelines provide well-defined—albeit contradictory—recommendations on how long elective procedures should be delayed following coronary stent placement. However, for patients needing urgent surgery, while still presumably requiring DAPT, the recommendations concerning antiplatelet medications become vague. Most of the guidelines simply advise somehow weighing the risk of stent thrombosis against the risk of bleeding and, if at all possible, continuing the DAPT therapy throughout the perioperative period. The most current ACC/AHA guidelines [14,19] do thoroughly review many of the risks associated with both stent thrombosis and surgical bleeding, but the incorporation of these risks into the final recommendations is incomplete.

### The Challenges of Implementing Professional Society Practice Guidelines

Developing such clinical practice guidelines is a highly-structured, labor intensive process, involving a rigorous review and critical appraisal of the literature, multidisciplinary consultation, and grading of the resulting recommendations based on the quality of available evidence [20]. Nevertheless, persistently variable success has been experienced in translating even well-grounded national clinical guidelines into local practice, including in the perioperative setting [21]. Barriers to providers adopting such clinical guidelines include inadequate understanding, lack of agreement and perceived “real-world” practicality, concerns about loss of self-efficacy, low outcome expectations, and the inertia of existing practice [22].

In an October 2010 survey of United States Department of Veterans Affairs network physicians, 100% of anesthesiologists, 100% of cardiologists, but only 78% of surgeons were aware of the then current ACC/AHA Guidelines on Perioperative Cardiovascular Evaluation and Care for Noncardiac Surgery [23]. Furthermore, among the survey respondents, 87% of anesthesiologists, 90% of cardiologists, but only 64% of surgeons agreed with these published guidelines. There was also significant variability among the three specialties in the perception of risk of coronary stent thrombosis versus bleeding and in the perioperative continuation of antiplatelet therapy with a BMS or a DES—with anesthesiologists and cardiologists emphasizing stent thrombosis risk and more often electing to continue antiplatelet therapy [23]. Anecdotally, at our institution, we had encountered minimal success in achieving consistent adherence to these same previously published ACC/AHA clinical practice guidelines for the preoperative management of patients with a coronary artery stent.

### The Creation of a Standardized Clinical Assessment and Management Plan for Pre-Procedural Antiplatelet Therapy in Patients with a Coronary Artery Stent

Neuman and colleagues at the University of Pennsylvania have observed that the durability of class I guideline recommendations for procedures and treatments, promulgated by the ACC/AHA, have varied widely across individual guidelines and levels of evidence [24]. Specifically, of 619 such ACC/AHA recommendations examined, 80.0% were retained in the subsequent guideline version, 9.2% were downgraded or reversed, and 10.8% were omitted. Downgrades, reversals, and omissions were most common among recommendations that were not supported by multiple randomized studies [24]. As noted in the accompanying editorial, in order for practice guidelines to be most effective, they need to be kept up-to-date [25].

The Institute of Medicine (IOM) has recommended that clinical practice guidelines should be updated when new evidence suggests the need for modification of such clinically important recommendations [26]. However, as noted in the above editorial: “But what does this mean, and how is it best accomplished?” [25]. To address this, three additional key questions should be considered: (1) When is there sufficient new evidence to “trigger” an update; (2) Once a trigger has been met, what methods can be used to expeditiously produce the update; (3) Once the update is completed, how is the information best communicated to relevant stakeholders? [25].

We posit that a standardized clinical assessment and management plan represents a viable response to the second and third key questions.

In contrast to professional society practice guidelines, a standardized clinical assessment and management plan (SCAMP) provides for a more “homegrown,” local clinician-designed and clinician-driven, yet rigorous alternative approach to achieving evidence-informed best practice for a heterogeneous patient population [27]. Clinicians are more likely to adopt practice guidelines that combine existing evidence and expert opinion in a format that can be readily revised. An iterative SCAMP hence accommodates local patients’ individual and population differences, respects local providers’ clinical acumen, and keeps pace with the rapid growth of medical knowledge [28]. For example, the published ACC/AHA guidelines, which were included in the above systematic review of perioperative management of antiplatelet therapy, date back to 2007/2009 [12,14]. These existing ACC/AHA guidelines are currently undergoing revision.

**Figure 1:**
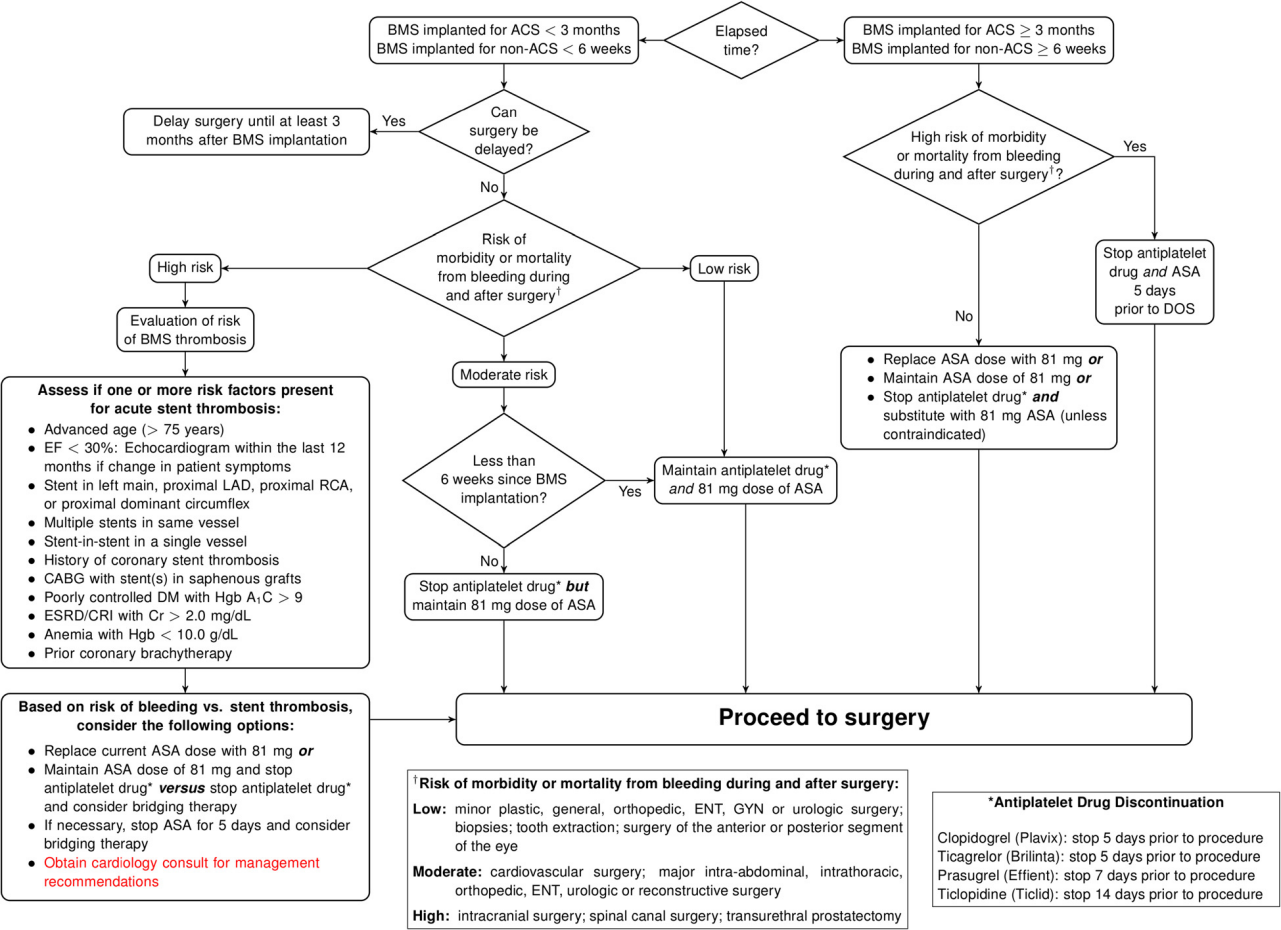
Protocol for Preoperative Antiplatelet Therapy with Bare Metal Stent (BMS).

**Figure 2:**
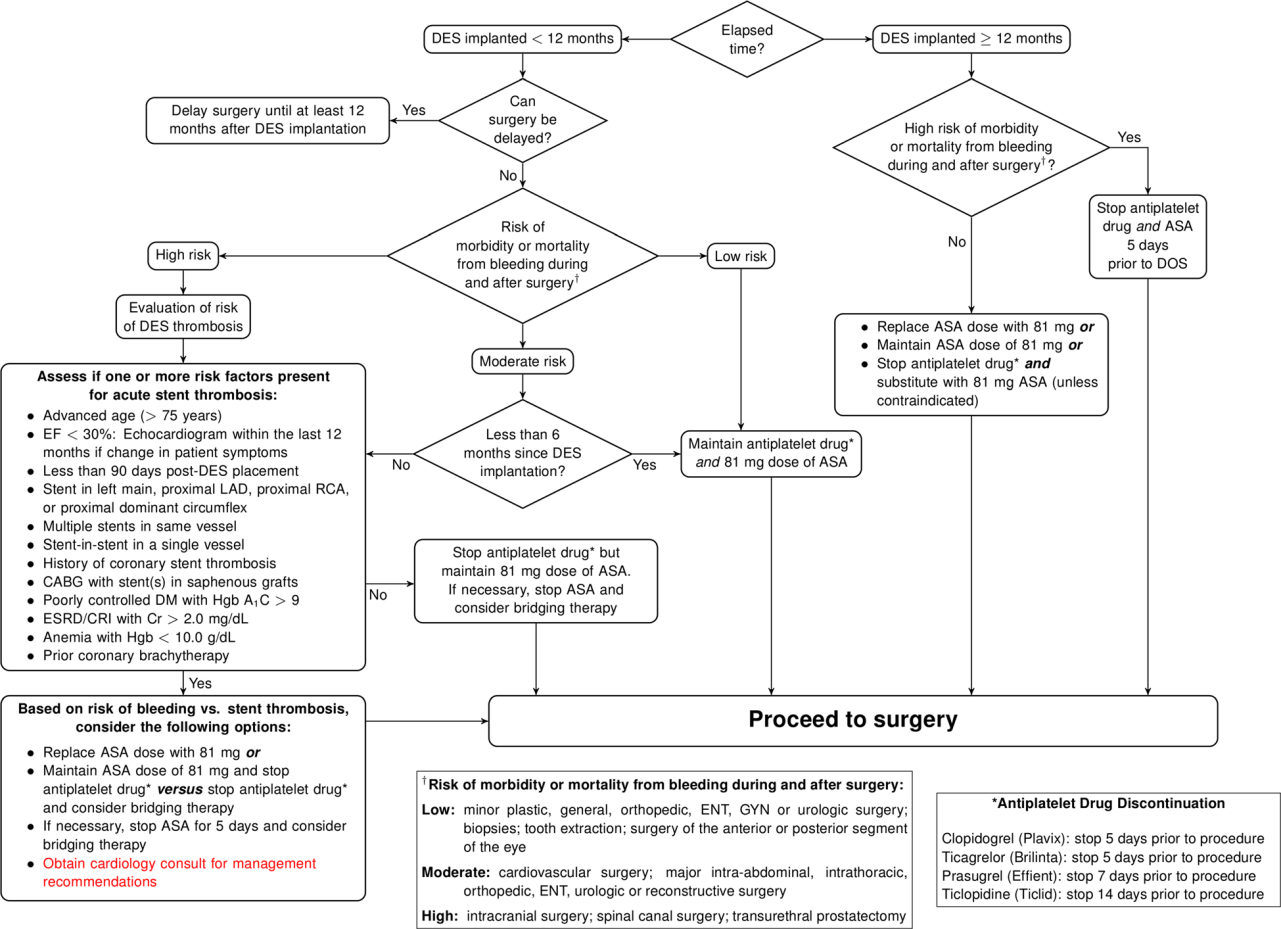
Protocol for Preoperative Antiplatelet Therapy with Drug-Eluting Stent (DES).

With this in mind, under the auspices of a broadly multidisciplinary Anticoagulation Task Force at the University of Alabama at Birmingham (List of Stakeholder), we created two current evidence-informed yet also local expert opinion-supported SCAMPs: one focused on preoperative antiplatelet therapy with a BMS (Figure 1) and one focused on preoperative antiplatelet therapy with a DES (Figure 2). In that this continuous quality improvement (CQI) project was not human subjects research, approval was not obtained from the Institutional Review Board of the University of Alabama at Birmingham.

### List of University of Alabama at Birmingham Health System Anticoagulation Task Force Stakeholder Members

Health System Chief of Staff

Anesthesiology

Cardiology

Critical Care Medicine

General Surgery

Hospitalist Medicine

Pain Medicine

Pathology

Pulmonary Medicine

Transfusion Medicine

Vascular Surgery

**Figure 3:**
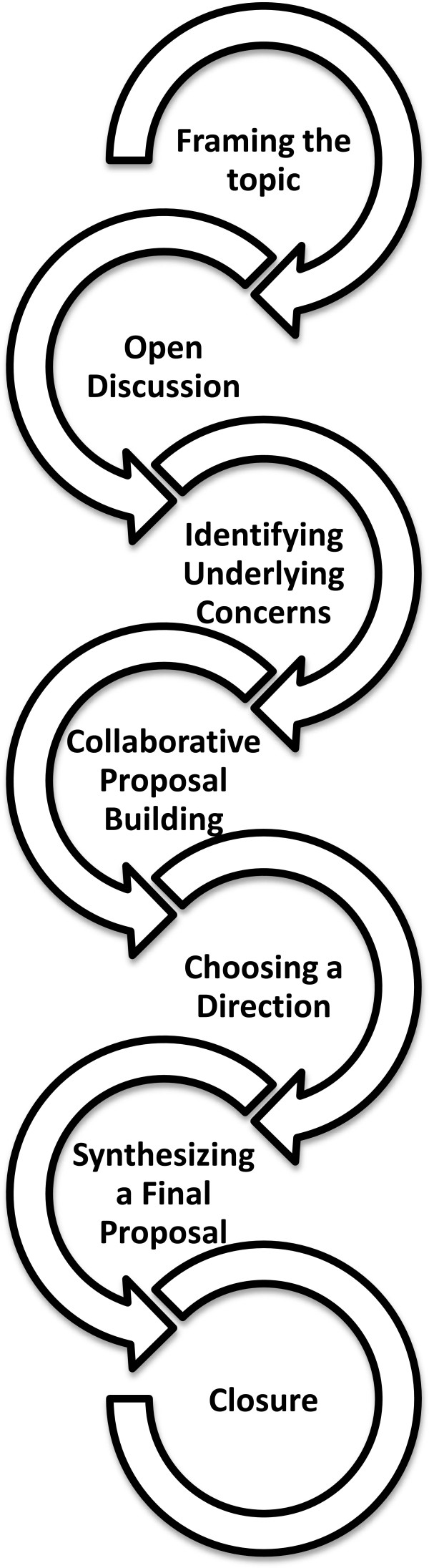
**The Seven Steps Involved in the Consensus-Oriented Decision-Making (CODM) model **[29]**.**

Over a 12-month period, our multidisciplinary anticoagulation task force applied the Consensus-Oriented Decision-Making (CODM) model to arrive at a consensus among the local clinical stakeholders. A detailed, step-wise process, the CODM model can be applied in any type of decision-making process (Figure 3). It outlines a process in which proposals can be collaboratively built with full participation of all stakeholders [29]. Consensus decision-making does not require unanimity but instead seeks the agreement of the majority of participants as well as the resolution or mitigation of minority held objection [30]. We took into consideration previously published applicable guidelines and other literature to create two evidence-informed protocols (see Additional file 1). These two protocols are presented here as *suggested* clinical management approaches.

## Discussion

It should be noted that our two present coronary artery SCAMPs are not evidence-based per se, pending additional published data. The central management principle that emerges from the present authors’ algorithms and the above existing guidelines [12,18] is the need to assess stent thrombotic risk (from preoperative withdrawal of antiplatelet therapy) *versus* bleeding risk (from perioperative continuation of antiplatelet therapy) for each surgical patient. Likewise, appropriate consideration is given to surgical procedures that are deemed urgent (e.g., cancer-related) and thus cannot be delayed until the time period of highest risk for acute coronary artery stent thrombosis has passed.

Our present recommendations provide greater clarity for the large majority of surgical patients. However, management of patients with a recently placed DES and risk factor(s) for acute stent thrombosis, undergoing intracranial or intraspinal surgery, remained controversial among our clinical stakeholders. In such situations, we advise that a formal Cardiology consult be obtained and bridging therapy be considered for such patients.

Once approved by our institutional medical executive committee (comprised primarily of our clinical departmental chairs, including all of our surgical departments), these SCAMPs were implemented health system-wide in January 2014, so as to provide standardized care to procedural patients with a BMS or DES. The clear stakeholder expectation is that these two SCAMPs will be promptly revised as pertinent new outcomes data or national organizational recommendations become available—again applying the CODM model.

Since their implementation only recently occurred, we do not have any clinical outcomes data to support the effectiveness of the two coronary artery stent SCAMPs at our institution. However, our planned quality assurance metrics include: 1) compliance rates with both SCAMPs; 2) number of surgical cases cancelled because of adherence and non-adherence to the applicable SCAMP; and 3) incidence of major bleeding events (including drop in hemoglobin below 8 g/dL, need for packed red blood cell or platelet transfusion) in patients on DAPT not discontinued prior to surgery; and 4) incidence of perioperative MACE.

## Summary

The need for continuing chronic antiplatelet therapy for coronary artery stents can be challenging and remains controversial in patients undergoing surgery. These challenges and controversies can best be addressed with an understanding of the pharmacology and applicable pharmacogenomics of antiplatelet drugs, continued evolution of the coronary artery stent, and pathophysiology and epidemiology of perioperative MACE with such stents [11]. Patient care can be optimized via evidence-based, yet locally developed and reiterative SCAMPs for patients with coronary artery stents undergoing surgical procedures. These patient-specific SCAMPs include not only the type of stent, indication, and elapsed time since placement, but also the stratified level of risk of morbidity and mortality from procedure-related bleeding. Such SCAMPs can result in greater consistency in care, providing a positive feedback loop in which the care plan itself can be continuously reevaluated, improved, and brought up to date with the most recent data and knowledge.

## Abbreviations

BMS: Bare metal stent; CODM: Consensus-Oriented Decision-Making; DAPT: Dual antiplatelet therapy; DES: Drug-eluting stent; MACE: Major adverse cardiac event; SCAMP: Standardized clinical assessment and management plan.

## Competing interests

All three authors declare that they have no financial, consultant, institutional or other relationships that resulted in bias or a conflict of interest in the conducting or reporting this study. The authors have no competing interests.

## Authors’ contributions

All three authors were involved in drafting the article and critically revising it for important coherency and intellectual content. TRV was responsible for organizing the overall manuscript, and specifically for the Abstract, Background, Summary, and the material on existing applicable guidelines and the challenges of implementing such practice guidelines. JMH was responsible for refining the two clinical care protocols. AMB was responsible for the material on standardized clinical assessment and management plans. All authors approved the final version to be submitted for publication.

## Authors’ information

TRV is the Maurice S. Albin Professor and Vice Chair of Pain Medicine in the Department of Anesthesiology at the University of Alabama at Birmingham. He is the Medical Director of the UAB Preoperative Assessment, Consultation, and Treatment Clinics.

JMH is an Assistant Professor in the Department of Anesthesiology at the University of Alabama at Birmingham. He is the Medical Director of the Intensive Care Unit at UAB Highlands Hospital.

AMB is a Professor and the Vice Chair for Quality and Patient Safety in the Department of Anesthesiology at the University of Alabama at Birmingham. He is the Chief of Staff of the UAB Health System.

## Pre-publication history

The pre-publication history for this paper can be accessed here:

http://www.biomedcentral.com/1471-2253/14/73/prepub

## Supplementary Material

Additional file 1 References Used for Developing Standardized Clinical Assessment and Management Plans for Preoperative Antiplatelet Therapy with a Bare Metal Stent and a Drug-Eluting Stent. Click here for file
