# Supplementary material for: Preoperative management of antiplatelet drugs for a coronary artery stent: how can we hit a moving target?
Source: BMC Anesthesiol. 2014 Aug 23;14:73. doi: 10.1186/1471-2253-14-73 (PMC4151077; doi:10.1186/1471-2253-14-73)
Supplement: Additional file 1 — References Used for Developing Standardized Clinical Assessment and Management Plans for Preoperative Antiplatelet Therapy with a Bare Metal Stent and a Drug-Eluting Stent. [file 1471-2253-14-73-S1.docx]

**Additional file**

**References Used for Developing Standardized Clinical Assessment and Management Plans for Preoperative Antiplatelet Therapy with a Bare Metal Stent and a Drug-Eluting Stent [**[**1-111**](#_ENREF_1)**].**

1. Abualsaud AO, Eisenberg MJ: **Perioperative management of patients with drug-eluting stents**. *JACC Cardiovasc Interv* 2010, **3**(2):131-142.

2. Albaladejo P, Marret E, Samama CM, Collet JP, Abhay K, Loutrel O, Charbonneau H, Jaber S, Thoret S, Bosson JL *et al*: **Non-cardiac surgery in patients with coronary stents: the RECO study**. *Heart* 2011, **97**(19):1566-1572.

3. Antolovic D, Reissfelder C, Rakow A, Contin P, Rahbari NN, Buchler MW, Weitz J, Koch M: **A randomised controlled trial to evaluate and optimize the use of antiplatelet agents in the perioperative management in patients undergoing general and abdominal surgery--the APAP trial (ISRCTN45810007)**. *BMC Surg* 2011, **11**:7.

4. Badreldin A, Kroener A, Kamiya H, Lichtenberg A, Hekmat K: **Effect of clopidogrel on perioperative blood loss and transfusion in coronary artery bypass graft surgery**. *Interact Cardiovasc Thorac Surg* 2010, **10**(1):48-52.

5. Barash P, Akhtar S: **Coronary stents: factors contributing to perioperative major adverse cardiovascular events**. *Br J Anaesth* 2010, **105 Suppl 1**:i3-15.

6. Bell AD, Roussin A, Cartier R, Chan WS, Douketis JD, Gupta A, Kraw ME, Lindsay TF, Love MP, Pannu N *et al*: **The use of antiplatelet therapy in the outpatient setting: Canadian Cardiovascular Society guidelines**. *Can J Cardiol* 2011, **27 Suppl A**:S1-59.

7. Bell AD, Roussin A, Cartier R, Chan WS, Douketis JD, Gupta A, Kraw ME, Lindsay TF, Love MP, Pannu N *et al*: **The use of antiplatelet therapy in the outpatient setting: Canadian Cardiovascular Society Guidelines Executive Summary**. *Can J Cardiol* 2011, **27**(2):208-221.

8. Bell B, Layland J, Poon K, Spaulding C, Walters D: **Focused clinical review: periprocedural management of antiplatelet therapy in patients with coronary stents**. *Heart Lung Circ* 2011, **20**(7):438-445.

9. Berger PB, Kleiman NS, Pencina MJ, Hsieh WH, Steinhubl SR, Jeremias A, Sonel A, Browne K, Barseness G, Cohen DJ: **Frequency of major noncardiac surgery and subsequent adverse events in the year after drug-eluting stent placement results from the EVENT (Evaluation of Drug-Eluting Stents and Ischemic Events) Registry**. *JACC Cardiovasc Interv* 2010, **3**(9):920-927.

10. Biondi-Zoccai GG, Lotrionte M, Agostoni P, Abbate A, Fusaro M, Burzotta F, Testa L, Sheiban I, Sangiorgi G: **A systematic review and meta-analysis on the hazards of discontinuing or not adhering to aspirin among 50,279 patients at risk for coronary artery disease**. *Eur Heart J* 2006, **27**(22):2667-2674.

11. Bornemann H, Pruller F, Metzler H: **The patient with coronary stents and antiplatelet agents: what to do and how to deal?** *Eur J Anaesthesiol* 2010, **27**(5):406-410.

12. Bracey AW, Reyes MA, Chen AJ, Bayat M, Allison PM: **How do we manage patients treated with antithrombotic therapy in the perioperative interval**. *Transfusion* 2011, **51**(10):2066-2077.

13. Brancati MF, Giammarinaro M, Burzotta F, Trani C, Coroleu SF, Porto I, Tommasino A, Leone AM, Niccoli G, Mongiardo R *et al*: **Outcome of non-cardiac surgery after stent implantation in the DES era: results of the Surgery After Stent (SAS) registry**. *J Invasive Cardiol* 2011, **23**(2):44-49.

14. Brilakis ES, Banerjee S, Berger PB: **Perioperative Management of Patients With Coronary Stents**. *J Am Coll Cardiol* 2007, **49**(22):2145-2150.

15. Broad L, Lee T, Conroy M, Bolsin S, Orford N, Black A, Birdsey G: **Successful management of patients with a drug-eluting coronary stent presenting for elective, non-cardiac surgery**. *Br J Anaesth* 2007, **98**(1):19-22.

16. Burger W, Chemnitius JM, Kneissl GD, Rucker G: **Low-dose aspirin for secondary cardiovascular prevention - cardiovascular risks after its perioperative withdrawal versus bleeding risks with its continuation - review and meta-analysis**. *J Intern Med* 2005, **257**(5):399-414.

17. Cassese S, Piccolo R, Galasso G, De Rosa R, Piscione F: **Twelve-month clinical outcomes of everolimus-eluting stent as compared to paclitaxel- and sirolimus-eluting stent in patients undergoing percutaneous coronary interventions. A meta-analysis of randomized clinical trials**. *Int J Cardiol* 2011, **150**(1):84-89.

18. Cerfolio RJ, Bryant AS: **The management of anticoagulants perioperatively**. *Thorac Surg Clin* 2012, **22**(1):29-34, v-vi.

19. Chassot PG, Delabays A, Spahn DR: **Perioperative antiplatelet therapy: the case for continuing therapy in patients at risk of myocardial infarction**. *Br J Anaesth* 2007, **99**(3):316-328.

20. Chassot PG, Delabays A, Spahn DR: **Perioperative use of anti-platelet drugs**. *Best Pract Res Clin Anaesthesiol* 2007, **21**(2):241-256.

21. Chassot PG, Marcucci C, Delabays A, Spahn DR: **Perioperative antiplatelet therapy**. *Am Fam Physician* 2010, **82**(12):1484-1489.

22. Chen TH, Matyal R: **The management of antiplatelet therapy in patients with coronary stents undergoing noncardiac surgery**. *Semin Cardiothorac Vasc Anesth* 2010, **14**(4):256-273.

23. Chou S, Eshaghian S, Lamer A, Tran H, Dohad S, Kaul S: **Bridging therapy in the perioperative management of patients with drug-eluting stents**. *Rev Cardiovasc Med* 2009, **10**(4):209-218.

24. Collet J-P, Montalescot G: **Premature withdrawal and alternative therapies to dual oral antiplatelet therapy**. *European Heart Journal Supplements* 2006, **8**(suppl G):G46-G52.

25. Collet JP, Aout M, Alantar A, Coriat P, Napoleon B, Thomas D, Trosini-Desert V, Tucas G, Vicaut E, Montalescot G: **Real-life management of dual antiplatelet therapy interruption: the REGINA survey**. *Arch Cardiovasc Dis* 2009, **102**(10):697-710.

26. Cruden NL, Harding SA, Flapan AD, Graham C, Wild SH, Slack R, Pell JP, Newby DE: **Previous coronary stent implantation and cardiac events in patients undergoing noncardiac surgery**. *Circ Cardiovasc Interv* 2010, **3**(3):236-242.

27. Dalal AR, D'Souza S, Shulman MS: **Brief review: coronary drug-eluting stents and anesthesia**. *Can J Anaesth* 2006, **53**(12):1230-1243.

28. Danielson D, Bjork K, Foreman J: **Preoperative evaluation**. *Institute for Clinical Systems Improvement* 2012, **10**:1-61.

29. Darvish-Kazem S, Douketis JD: **Perioperative management of patients having noncardiac surgery who are receiving anticoagulant or antiplatelet therapy: an evidence-based but practical approach**. *Semin Thromb Hemost* 2012, **38**(7):652-660.

30. Darvish-Kazem S, Gandhi M, Marcucci M, Douketis JD: **Perioperative management of antiplatelet therapy in patients with a coronary stent who need noncardiac surgery: a systematic review of clinical practice guidelines**. *Chest* 2013, **144**(6):1848-1856.

31. Dimitrova G, Tulman DB, Bergese SD: **Perioperative management of antiplatelet therapy in patients with drug-eluting stents**. *HSR Proc Intensive Care Cardiovasc Anesth* 2012, **4**(3):153-167.

32. Dineen PF, Curtin RJ, Harty JA: **A review of the use of common antiplatelet agents in orthopaedic practice**. *J Bone Joint Surg Br* 2010, **92**(9):1186-1191.

33. Douketis JD: **Perioperative management of patients receiving anticoagulant or antiplatelet therapy: a clinician-oriented and practical approach**. *Hosp Pract (Minneap)* 2011, **39**(4):41-54.

34. Douketis JD, Spyropoulos AC, Spencer FA, Mayr M, Jaffer AK, Eckman MH, Dunn AS, Kunz R: **Perioperative management of antithrombotic therapy: antithrombotic therapy and prevention of thrombosis, 9th ed: American College of Chest Physicians evidence-based clinical practice guidelines**. *Chest* 2012, **141**(2 Suppl):e326S-350S.

35. Eng M, Brock G, Li X, Chen Y, Ravindra KV, Buell JF, Marvin MR: **Perioperative anticoagulation and antiplatelet therapy in renal transplant: is there an increase in bleeding complication?** *Clin Transplant* 2011, **25**(2):292-296.

36. Engelen S, Sinnaeve P, Van Damme H, Verhamme P: **Antiplatelet therapy in the perioperative period**. *Acta Anaesthesiol Belg* 2010, **61**(3):139-143.

37. Farb A, Boam AB: **Stent thrombosis redux--the FDA perspective**. *N Engl J Med* 2007, **356**(10):984-987.

38. Ferrari E, Benhamou M, Cerboni P, Marcel B: **Coronary syndromes following aspirin withdrawal: a special risk for late stent thrombosis**. *J Am Coll Cardiol* 2005, **45**(3):456-459.

39. Ferraris VA, Saha SP, Oestreich JH, Song HK, Rosengart T, Reece TB, Mazer CD, Bridges CR, Despotis GJ, Jointer K *et al*: **2012 update to the Society of Thoracic Surgeons guideline on use of antiplatelet drugs in patients having cardiac and noncardiac operations**. *Ann Thorac Surg* 2012, **94**(5):1761-1781.

40. Fleisher LA, Beckman JA, Brown KA, Calkins H, Chaikof EL, Fleischmann KE, Freeman WK, Froehlich JB, Kasper EK, Kersten JR *et al*: **2009 ACCF/AHA focused update on perioperative beta blockade incorporated into the ACC/AHA 2007 guidelines on perioperative cardiovascular evaluation and care for noncardiac surgery: a report of the American college of cardiology foundation/American heart association task force on practice guidelines**. *Circulation* 2009, **120**(21):e169-276.

41. Gallego P, Apostolakis S, Lip GYH: **Bridging evidence-based practice and practice-based evidence in periprocedural anticoagulation**. *Circulation* 2012, **126**(13):1573-1576.

42. Gandhi NK, Abdel-Karim AR, Banerjee S, Brilakis ES: **Frequency and risk of noncardiac surgery after drug-eluting stent implantation**. *Catheter Cardiovasc Interv* 2011, **77**(7):972-976.

43. Gerstein NS, Schulman PM, Gerstein WH, Petersen TR, Tawil I: **Should more patients continue aspirin therapy perioperatively?: clinical impact of the aspirin withdrawal syndrome**. *Ann Surg* 2012.

44. Gibbs NM: **Point-of-care assessment of antiplatelet agents in the perioperative period: a review**. *Anaesth Intensive Care* 2009, **37**(3):354-369.

45. Grines CL, Bonow RO, Casey DE, Jr., Gardner TJ, Lockhart PB, Moliterno DJ, O'Gara P, Whitlow P: **Prevention of premature discontinuation of dual antiplatelet therapy in patients with coronary artery stents: a science advisory from the American Heart Association, American College of Cardiology, Society for Cardiovascular Angiography and Interventions, American College of Surgeons, and American Dental Association, with representation from the American College of Physicians**. *Circulation* 2007, **115**(6):813-818.

46. Gulbins H, Malkoc A, Ennker IC, Ennker J: **Preoperative platelet inhibition with ASA does not influence postoperative blood loss following coronary artery bypass grafting**. *Thorac Cardiovasc Surg* 2009, **57**(1):18-21.

47. Hall R, Mazer CD: **Antiplatelet drugs: a review of their pharmacology and management in the perioperative period**. *Anesth Analg* 2011, **112**(2):292-318.

48. Harjai KJ, Shenoy C, Orshaw P, Usmani S, Singh M, Boura J, Mehta RH: **Low-dose versus high-dose aspirin after percutaneous coronary intervention: Analysis from the guthrie health off-label StenT (GHOST) registry**. *J Interv Cardiol* 2011, **24**(4):307-314.

49. Heir JS, Gottumukkala V, Singh M, Yusuf SW, Riedel B: **Coronary stents and noncardiac surgery: current clinical challenges and conundrums**. *Prev Cardiol* 2010, **13**(1):8-13.

50. Ho PM, Peterson ED, Wang L, Magid DJ, Fihn SD, Larsen GC, Jesse RA, Rumsfeld JS: **Incidence of death and acute myocardial infarction associated with stopping clopidogrel after acute coronary syndrome**. *JAMA* 2008, **299**(5):532-539.

51. Ho PM, Tsai TT, Wang TY, Shetterly SM, Clarke CL, Go AS, Sedrakyan A, Rumsfeld JS, Peterson ED, Magid DJ: **Adverse events after stopping clopidogrel in post-acute coronary syndrome patients: insights from a large integrated healthcare delivery system**. *Circ Cardiovasc Qual Outcomes* 2010, **3**(3):303-308.

52. Hollis RH, Graham LA, Richman JS, Deierhoi RJ, Hawn MT: **Adverse cardiac events in patients with coronary stents undergoing noncardiac surgery: a systematic review**. *Am J Surg* 2012, **204**(4):494-501.

53. Howard-Alpe GM, de Bono J, Hudsmith L, Orr WP, Foex P, Sear JW: **Coronary artery stents and non-cardiac surgery**. *Br J Anaesth* 2007, **98**(5):560-574.

54. Iakovou I, Schmidt T, Bonizzoni E, Ge L, Sangiorgi GM, Stankovic G, Airoldi F, Chieffo A, Montorfano M, Carlino M *et al*: **Incidence, predictors, and outcome of thrombosis after successful implantation of drug-eluting stents**. *JAMA* 2005, **293**(17):2126-2130.

55. Kaatz S, Paje D: **Update in bridging anticoagulation**. *Journal of Thrombosis and Thrombolysis* 2011, **31**(3):259-264.

56. Kaatz S, Paje DG: **Perioperative management of anticoagulation**. In: *Perioperative Medicine.* edn. Edited by Cohn SL. London: Springer; 2011: 45-53.

57. Kennedy MT, Roche S, Fleming SM, Lenehan B, Curtin W: **The association between aspirin and blood loss in hip fracture patients**. *Acta Orthop Belg* 2006, **72**(1):29-33.

58. Khair T, Garcia B, Banerjee S, Brilakis ES: **Contemporary approaches to perioperative management of coronary stents and to preoperative coronary revascularization: A survey of 374 interventional cardiologists**. *Cardiovasc Revasc Med* 2011, **12**(2):99-104.

59. Kim DH, Daskalakis C, Silvestry SC, Sheth MP, Lee AN, Adams S, Hohmann S, Medvedev S, Whellan DJ: **Aspirin and clopidogrel use in the early postoperative period following on-pump and off-pump coronary artery bypass grafting**. *J Thorac Cardiovasc Surg* 2009, **138**(6):1377-1384.

60. King SB, 3rd, Smith SC, Jr., Hirshfeld JW, Jr., Jacobs AK, Morrison DA, Williams DO, Feldman TE, Kern MJ, O'Neill WW, Schaff HV *et al*: **2007 focused update of the ACC/AHA/SCAI 2005 guideline update for percutaneous coronary intervention: A report of the American College of Cardiology/American Heart Association Task Force on Practice Guidelines: 2007 Writing Group to Review New Evidence and Update the ACC/AHA/SCAI 2005 guideline update for percutaneous coronary intervention, writing on behalf of the 2005 Writing Committee**. *Circulation* 2008, **117**(2):261-295.

61. Korte W, Cattaneo M, Chassot PG, Eichinger S, von Heymann C, Hofmann N, Rickli H, Spannagl M, Ziegler B, Verheugt F *et al*: **Peri-operative management of antiplatelet therapy in patients with coronary artery disease: joint position paper by members of the working group on Perioperative Haemostasis of the Society on Thrombosis and Haemostasis Research (GTH), the working group on Perioperative Coagulation of the Austrian Society for Anesthesiology, Resuscitation and Intensive Care (OGARI) and the Working Group Thrombosis of the European Society for Cardiology (ESC)**. *Thromb Haemost* 2011, **105**(5):743-749.

62. Kraai E, Lopes R, Alexander J, Garcia D: **Perioperative management of anticoagulation: guidelines translated for the clinician**. *Journal of Thrombosis and Thrombolysis* 2009, **28**(1):16-22.

63. Lanckohr C, Torsello G, Scheld H, Schieffer B, Theilmeier G: **Drug-eluting stents and perioperative risk - more than matters of the heart?** *Vasa* 2012, **41**(6):410-418.

64. Langton TJ, Walker JL: **Perioperative management of the patient with coronary stents**. *Int Anesthesiol Clin* 2011, **49**(2):20-25.

65. Llau JV, Ferrandis R, Sierra P, Gomez-Luque A: **Prevention of the renarrowing of coronary arteries using drug-eluting stents in the perioperative period: an update**. *Vasc Health Risk Manag* 2010, **6**:855-867.

66. Llau JV, Lopez-Forte C, Sapena L, Ferrandis R: **Perioperative management of antiplatelet agents in noncardiac surgery**. *Eur J Anaesthesiol* 2009, **26**(3):181-187.

67. Luckie M, Khattar RS, Fraser D: **Non-cardiac surgery and antiplatelet therapy following coronary artery stenting**. *Heart* 2009, **95**(16):1303-1308.

68. Luscher TF, Steffel J, Eberli FR, Joner M, Nakazawa G, Tanner FC, Virmani R: **Drug-eluting stent and coronary thrombosis: biological mechanisms and clinical implications**. *Circulation* 2007, **115**(8):1051-1058.

69. Mantz J, Dahmani S, Paugam-Burtz C: **Outcomes in perioperative care**. *Curr Opin Anaesthesiol* 2010, **23**(2):201-208.

70. Mantz J, Samama CM, Tubach F, Devereaux PJ, Collet JP, Albaladejo P, Cholley B, Nizard R, Barre J, Piriou V *et al*: **Impact of preoperative maintenance or interruption of aspirin on thrombotic and bleeding events after elective non-cardiac surgery: the multicentre, randomized, blinded, placebo-controlled, STRATAGEM trial**. *Br J Anaesth* 2011, **107**(6):899-910.

71. May AE, Geisler T, Gawaz M: **Individualized antithrombotic therapy in high risk patients after coronary stenting. A double-edged sword between thrombosis and bleeding**. *Thromb Haemost* 2008, **99**(3):487-493.

72. Metzler H, Kozek-Langenecker S, Huber K: **Antiplatelet therapy and coronary stents in perioperative medicine--the two sides of the coin**. *Best Pract Res Clin Anaesthesiol* 2008, **22**(1):81-94.

73. Nef HM, Mollmann H, Hamm CW: **Anticoagulation in percutaneous coronary interventions: no man's land?** *Eur Heart J* 2010, **31**(20):2447-2448.

74. Newsome LT, Kutcher MA, Royster RL: **Coronary artery stents: Part I. Evolution of percutaneous coronary intervention**. *Anesth Analg* 2008, **107**(2):552-569.

75. Newsome LT, Weller RS, Gerancher JC, Kutcher MA, Royster RL: **Coronary artery stents: II. Perioperative considerations and management**. *Anesth Analg* 2008, **107**(2):570-590.

76. Niemi T, Armstrong E: **Thromboprophylactic management in the neurosurgical patient with high risk for both thrombosis and intracranial bleeding**. *Curr Opin Anaesthesiol* 2010, **23**(5):558-563.

77. Nuttall GA, Brown MJ, Stombaugh JW, Michon PB, Hathaway MF, Lindeen KC, Hanson AC, Schroeder DR, Oliver WC, Holmes DR *et al*: **Time and cardiac risk of surgery after bare-metal stent percutaneous coronary intervention**. *Anesthesiology* 2008, **109**(4):588-595.

78. O'Riordan JM, Margey RJ, Blake G, O'Connell PR: **Antiplatelet agents in the perioperative period**. *Arch Surg* 2009, **144**(1):69-76; discussion 76.

79. Oscarsson A, Gupta A, Fredrikson M, Jarhult J, Nystrom M, Pettersson E, Darvish B, Krook H, Swahn E, Eintrei C: **To continue or discontinue aspirin in the perioperative period: a randomized, controlled clinical trial**. *Br J Anaesth* 2010, **104**(3):305-312.

80. Ovrum E, Tangen G, Tollofsrud S, Ringdal MA, Oystese R, Istad R: **Low postoperative dose of aprotinin reduces bleeding and is safe in patients receiving clopidogrel before coronary artery bypass surgery. A prospective randomized study**. *Interact Cardiovasc Thorac Surg* 2010, **10**(4):545-548.

81. Palareti G: **Bridging therapy in oral anticoagulated patients: focusing on how to do it**. *Internal and Emergency Medicine* 2007, **2**(4):247-249.

82. Palmerini T, Biondi-Zoccai G, Della Riva D, Stettler C, Sangiorgi D, D'Ascenzo F, Kimura T, Briguori C, Sabate M, Kim HS *et al*: **Stent thrombosis with drug-eluting and bare-metal stents: evidence from a comprehensive network meta-analysis**. *Lancet* 2012, **379**(9824):1393-1402.

83. Parikh A, Toepfer N, Baylor K, Henry Y, Berger P, Rukstalis D: **Preoperative aspirin is aafe in patients undergoing urologic robot-assisted surgery**. *J Endourol* 2012.

84. Peter K, Myles PS: **Perioperative antiplatelet therapy: A knife-edged choice between thrombosis and bleeding still based on consensus rather than evidence**. *Thromb Haemost* 2011, **105**(5):750-751.

85. Poldermans D, Bax JJ, Boersma E, De Hert S, Eeckhout E, Fowkes G, Gorenek B, Hennerici MG, Iung B, Kelm M *et al*: **Guidelines for pre-operative cardiac risk assessment and perioperative cardiac management in non-cardiac surgery: The Task Force for Preoperative Cardiac Risk Assessment and Perioperative Cardiac Management in Non-cardiac Surgery of the European Society of Cardiology (ESC) and endorsed by the European Society of Anaesthesiology (ESA)**. *Eur J Anaesthesiol* 2010, **27**(2):92-137.

86. Popescu WM: **Perioperative management of the patient with a coronary stent**. *Curr Opin Anaesthesiol* 2010, **23**(1):109-115.

87. Price J, Tadbiri S: **Aspirin and clopidogrel use in the perioperative period for non-cardiac surgery**. *Br J Hosp Med (Lond)* 2008, **69**(1):56.

88. Rabbitts JA, Nuttall GA, Brown MJ, Hanson AC, Oliver WC, Holmes DR, Rihal CS: **Cardiac risk of noncardiac surgery after percutaneous coronary intervention with drug-eluting stents**. *Anesthesiology* 2008, **109**(4):596-604.

89. Raber L, Windecker S: **Current status of drug-eluting stents**. *Cardiovasc Ther* 2011, **29**(3):176-189.

90. Rade JJ, Hogue CW, Jr.: **Noncardiac surgery for patients with coronary artery stents: timing is everything**. *Anesthesiology* 2008, **109**(4):573-575.

91. Riddell JW, Chiche L, Plaud B, Hamon M: **Coronary stents and noncardiac surgery**. *Circulation* 2007, **116**(16):e378-382.

92. Roth E, Purnell C, Shabalov O, Moguillansky D, Hernandez CA, Elnicki M: **Perioperative management of a patient with recently placed drug-eluting stents requiring urgent spinal surgery**. *J Gen Intern Med* 2012.

93. Sambu N, Warner T, Curzen N: **Clopidogrel withdrawal: is there a "rebound" phenomenon?** *Thromb Haemost* 2011, **105**(2):211-220.

94. Savonitto S, Caracciolo M, Cattaneo M, S DES: **Management of patients with recently implanted coronary stents on dual antiplatelet therapy who need to undergo major surgery**. *J Thromb Haemost* 2011, **9**(11):2133-2142.

95. Schouten O, Bax JJ, Poldermans D: **Management of patients with cardiac stents undergoing noncardiac surgery**. *Curr Opin Anaesthesiol* 2007, **20**(3):274-278.

96. Servin F: **Low-dose aspirin and clopidogrel: How to act in patients scheduled for day surgery**. *Curr Opin Anaesthesiol* 2007, **20**(6):531-534.

97. Servin FS: **Is it time to re-evaluate the routines about stopping/keeping platelet inhibitors in conjunction to ambulatory surgery?** *Curr Opin Anaesthesiol* 2010, **23**(6):691-696.

98. Sharma AK, Ajani AE, Hamwi SM, Maniar P, Lakhani SV, Waksman R, Lindsay J: **Major noncardiac surgery following coronary stenting: when is it safe to operate?** *Catheter Cardiovasc Interv* 2004, **63**(2):141-145.

99. Singla S, Sachdeva R, Uretsky BF: **The risk of adverse cardiac and bleeding events following noncardiac surgery relative to antiplatelet therapy in patients with prior percutaneous coronary intervention**. *J Am Coll Cardiol* 2012, **60**(20):2005-2016.

100. So D, Cook EF, Le May M, Glover C, Williams W, Ha A, Davies R, Froeschl M, Marquis JF, O'Brien E *et al*: **Association of aspirin dosage to clinical outcomes after percutaneous coronary intervention: observations from the Ottawa Heart Institute PCI Registry**. *J Invasive Cardiol* 2009, **21**(3):121-127.

101. Spahn DR, Howell SJ, Delabays A, Chassot PG: **Coronary stents and perioperative anti-platelet regimen: dilemma of bleeding and stent thrombosis**. *Br J Anaesth* 2006, **96**(6):675-677.

102. Stone DH, Goodney PP, Schanzer A, Nolan BW, Adams JE, Powell RJ, Walsh DB, Cronenwett JL: **Clopidogrel is not associated with major bleeding complications during peripheral arterial surgery**. *J Vasc Surg* 2011, **54**(3):779-784.

103. Tandar A, Velagapudi KN, Wilson BD, Boden WE: **Perioperative antiplatelet management in patients with coronary artery stenting**. *Hosp Pract (1995)* 2012, **40**(2):118-130.

104. Thachil J, Gatt A, Martlew V: **Management of surgical patients receiving anticoagulation and antiplatelet agents**. *Br J Surg* 2008, **95**(12):1437-1448.

105. To AC, Armstrong G, Zeng I, Webster MW: **Noncardiac surgery and bleeding after percutaneous coronary intervention**. *Circ Cardiovasc Interv* 2009, **2**(3):213-221.

106. Vaclavik J, Taborsky M: **Antiplatelet therapy in the perioperative period**. *Eur J Intern Med* 2011, **22**(1):26-31.

107. Vandvik PO, Lincoff AM, Gore JM, Gutterman DD, Sonnenberg FA, Alonso-Coello P, Akl EA, Lansberg MG, Guyatt GH, Spencer FA: **Primary and secondary prevention of cardiovascular disease: antithrombotic therapy and prevention of thrombosis, 9th ed: American College of Chest Physicians evidence-based clinical practice guidelines**. *Chest* 2012, **141**(2 Suppl):e637S-668S.

108. Weitzel NS, Edelstein SB, Cleveland JC, Jr., Cornelissen CB: **Drug-eluting stents in the perioperative period: what are the key aspects in management?** *Semin Cardiothorac Vasc Anesth* 2011, **15**(1-2):44-48.

109. Wijeysundera DN, Wijeysundera HC, Yun L, Wasowicz M, Beattie WS, Velianou JL, Ko DT: **Risk of elective major noncardiac surgery after coronary stent insertion: a population-based study**. *Circulation* 2012, **126**(11):1355-1362.

110. Wilson SH, Fasseas P, Orford JL, Lennon RJ, Horlocker T, Charnoff NE, Melby S, Berger PB: **Clinical outcome of patients undergoing non-cardiac surgery in the two months following coronary stenting**. *J Am Coll Cardiol* 2003, **42**(2):234-240.

111. Yan BP, Gurvitch R, Ajani AE: **Double jeopardy: balance between bleeding and stent thrombosis with prolonged dual antiplatelet therapy after drug-eluting stent implantation**. *Cardiovasc Revasc Med* 2006, **7**(3):155-158.
